# Exosomal MiR-769-5p Exacerbates Ultraviolet-Induced Bystander Effect by Targeting *TGFBR1*

**DOI:** 10.3389/fphys.2020.603081

**Published:** 2020-11-23

**Authors:** Na Ni, Weiwei Ma, Yanling Tao, Juan Liu, Hui Hua, Jiawei Cheng, Jie Wang, Bingrong Zhou, Dan Luo

**Affiliations:** Department of Dermatology, The First Affiliated Hospital of Nanjing Medical University, Nanjing, China

**Keywords:** miRNA, exosomes, *TGFBR1*, ultraviolet–radiation induced bystander effects, photo damage

## Abstract

Exosomal microRNAs have been investigated in bystander effect, but it is unclear whether microRNA works in ultraviolet radiation–induced bystander effects (UV-RIBEs) and what the underlying mechanism could be. Exosomes from ultraviolet (UV)–irradiated human skin fibroblasts (HSFs) were isolated and transferred to normal HSFs, followed by the detection of proliferation rate, oxidative damage level, and apoptosis rate. Exosomal miRNAs were evaluated and screened with miRNA sequencing and quantitative reverse transcriptase–polymerase chain reaction method. MiRNA shuttle and bystander photodamage reactions were observed after transfection of miR-769-5p. MiR-769-5p targeting gene transforming growth factor-β1 (*TGFBR1*), and *TGFBR1* mRNA 3′-untranslated region (UTR) was assessed and identified by Western blotting and dual-luciferase reporter assay. Bystander effects were induced after being treated with isolated exosomes from UV-irradiated HSFs. Exosomal miR-769-5p expression was significantly upregulated. Human skin fibroblasts showed lower proliferation, increasing oxidative damage, and faster occurrence of apoptosis after transfection. Exosome-mediated transfer of miR-769-5p was observed. Upregulation of miR-769-5p induced bystander effects, whereas downregulation of miR-769-5p can suppress UV-RIBEs. In addition, miR-769-5p was found to downregulate *TGFBR1* gene expression by directly targeting its 3′-UTR. Our results demonstrate that exosome-mediated miR-769-5p transfer could function as an intercellular messenger and exacerbate UV-RIBEs. MiR-769-5p inhibits the expression of *TGFBR1* by targeting *TGFBR1* mRNA 3′-UTR.

## Introduction

Ultraviolet (UV) radiation is a kind of natural source of radiation, causing cutaneous photodamage and progressive injured changes in organisms (Davinelli et al., 2018). In previous researches, besides inducing direct damage in irradiated cells, radiation can also cause damage in adjacent non-irradiated cells after receiving signals, which is called radiation-induced bystander effects (RIBEs; Lin et al., 2017). Similar phenomenon can be observed in UV radiation as well (Eftekhari and Fardid, 2019). Krzywon and Widel (2019) found that viability of non-irradiated cells would be reduced when coincubated with irradiated cells. Induction of cell-cycle arrest at the G2/M transition was observed in non-irradiated cells treated with conditioned medium from UVC-irradiated A375 human melanoma cells (Ghosh et al., 2013). Widel et al. (2014) took a research on UV radiation–induced bystander effects (UV-RIBEs) by the method of Transwell coincubation system and found that UVA and UVB were more effective in inducing apoptosis of bystander cells than UVC. Although the phenomenon has been observed in plenty of experiments, the underlying causes of bystander effect are still poorly understood.

Recent researches increasingly emphasized the role of exosome in intercellular communication. Exosomes are a population of vesicles containing lipids, proteins, and nucleic acids, with a diameter of 20–200 nm, released by budding of the plasma membrane and involved in various biological events through delivering their cargos to exosome-receiving cells (Farooqi et al., 2018). Non-coding RNAs transported through exosomes, such as miRNA, siRNA, and piRNA, can combine with specific complementary sequences of target mRNAs and act as negative regulators (Najafi et al., 2014; Balaguer et al., 2019). MiRNAs have been researched over the past decades and found to express differentially in various diseases. Kura et al. (2019) proved that miRNA-1, -15b, and -21 may take part in protection of molecular hydrogen in irradiation-induced heart damage. Cavallari et al. (2019) demonstrated that miR-223 induced dysfunction of endothelial cells and vascular smooth muscle cells. Meanwhile, miRNAs packed into exosomes were reported to play important roles in bystander effect, functioning as intercellular transmitters from donor cells to recipient cells, altering biological processes such as terminal differentiation, cell cycle, apoptosis, and DNA damage responses in recipient bystander cells (Penfornis et al., 2016; Le et al., 2017). For example, Xu et al. (2014) indicated that miR-21 was involved in intercellular communication and influenced ionizing RIBEs consequently. MiR-663 has been found to be an inhibitor of bystander signal transmission by suppressing the expression of TGF-β1 (Hu et al., 2014). During the maturation of miRNAs, a sequence of significant steps is contained including long primary transcripts (pri-miRNAs) into stem-loop precursors (pre-miRNAs) and pre-miRNAs into mature miRNAs (Lee et al., 2003). Although miRNAs draw growing concern in recent years, with a set of researches on miRNAs in bystander effects, there is still a lack of researches about exosome-mediated miRNAs shuttle participating in UV-RIBEs. The mechanisms in miRNAs still need to be further investigated.

In this study, UV-RIBEs models were established by exosome transfer, after which differential expressed miRNAs were detected with miRNA microarray technology and quantitative reverse transcriptase–polymerase chain reaction (qRT-PCR) method. Expression of miR-769-5p was found to be increased significantly, and its exosome-mediated shuttle was tracked by fluorescence microscopy. We tested biological indicators of miR-769-5p transfected human skin fibroblasts (HSFs) and bystander HSFs after transfer of exosomes. In addition, the miR-769-5p targeting sequence transforming growth factor-β1 (*TGFBR1*) was verified with a dual-luciferase reporter assay. Our study further explored UV-RIBEs *in vitro* and found miR-769-5p as an important role. Exosomal miR-769-5p shuttle was observed and its relative signaling pathway was investigated.

## Materials and Methods

### Cell Culture and UV Irradiation

Primary HSFs were obtained from donors by means of a foreskin circumcision. Human skin fibroblasts were routinely cultured in either tissue culture flasks or well plates at 37°C in an atmosphere of 95% air and 5% CO_2_. Dulbecco modified eagle medium (DMEM) with 10% fetal bovine serum (FBS) (Hyclone, United States), 100 U/mL penicillin, and 100 mg/mL streptomycin was supplied to promote cell growth. The dose of UVA and UVB referred to preliminary experiments. UV irradiation was generated by UV phototherapy instruments (Sigma, Shanghai, China), whereas irradiation output was measured with irradiance monitor (Sigma, Shanghai, China). Human skin fibroblasts were covered with phosphate-buffered saline (PBS) during irradiation, which was removed immediately after irradiation. Then, HSFs were covered by culture medium with 10% FBS again. In order to remove bovine exosomes, we collected the supernatant carefully after centrifuging it at 100,000 *g*, 4°C for 1 h, and then repeated the steps for another time. The collected supernatant was filtered in centrifugal filters at 5,000 *g*, 4°C for 30 min. We retrieved the liquid, filtrated, and removed bacteria using 0.22-μm membrane filter.

### Extraction and Identification of Exosomes

After 24 h of different treatment, the exosomes from the conditioned medium were obtained through differential centrifugation. Briefly, the medium was collected and centrifuged at 500 *g* for 20 min, 1500 *g* for 20 min, and 10,000 *g* for 5 min at 4°C to remove cells, impurities, and other microvesicles and centrifuged at 100,000 *g* for 70 min to obtain pelleted exosomes.

The exosome pellet was resuspended with moderate PBS. The exosomes isolated was identified by Western blotting (WB) for detection the marker proteins of exosomes, transmission electron microscopy (TEM) observing the morphology and the size, and nanoparticle tracking analysis (NTA) for detecting the practical size and distribution of exosomes. Besides, the exosomes-free FBS was prepared through differential centrifugation described as above, and DMEM containing only 10% FBS without exosomes was prepared for cultivating HSFs. Exosomes extracted from the supernatant of each 15-cm culture dish were resuspended in 10 μL PBS, which is boiled for use after adding 5 × sodium dodecyl sulfate (SDS) loading buffer. The specific steps of WB will be explained in detail below. The exosomes were extracted according to step above and resuspended in moderate PBS. Then, 10 μL of the sample was aspirated and added to the copper wire mesh for 1 min, and the filter paper was taken up to remove the floating liquid.

Two percent uranyl acetate 10 μL was pipetted into the copper wire mesh for 1 min, and the floating liquid was removed as before. After drying for 15 min at room temperature, the morphology and size of the exosomes were observed on the machine. The exosomes resuspended with PBS were analyzed with NTA (ZetaView) and repeated three times.

### Exosomes Transfer Experiments

Exosomes were marked with PKH26 according to the manufacturer’s instruction (Sigma, Germany). Labeled exosomes were co-cultured with normal HSFs with exosomes-free medium when the cells were at 30% confluence. After 48-h culture, the cells were washed with PBS and fixed with 4% paraformaldehyde. Then, the cells were washed three times with PBS containing 0.1% Triton X-100 for 5 min each time. Actin-Tracker Green with immunofluorescent staining secondary antibody was diluted in a ratio of 1:100, where the cells were incubated at room temperature for 30 min in the dark (cell skeleton stained). DAPI staining solution was added after washing the cells with PBS containing 0.1% Triton X-100. After incubation for 30 min, the staining solution was discarded, and cells were observed under a confocal microscope.

### Cell Proliferation Assay

Human skin fibroblasts were uniformly scattered in 96-well plates with the density of 2000/100 μL. Culture medium was replaced by medium containing 10% cell counting kit-8 (CCK-8) colorimetric assay (Dojindo, Japan) after different managements were done for 48 h. Optical density (OD) values were measured at 450 nm by colorimetry spectrophotometry after 2-h incubation.

### 5-Ethynyl-2-Deoxyuridine Staining for Evaluation of Proliferative Activity

5-Ethynyl-2-deoxyuridine (EdU) reagent (RiboBio, Guangzhou, China) was diluted with medium in a ratio of 1000:1. Forty-eight hours after the last treatment, the EdU dilution was added to each group of HSFs for an additional 2 h, respectively. Then, the cells were fixed with PBS containing 4% paraformaldehyde after being washed with PBS. Then, the cells were treated with 2 mg/mL glycine and then washed as above. The cells were permeabilized with PBS containing 0.5% triton X-100. Cells were incubated with 1 × Apollo reaction cocktail (300 μL/well) for 30 min and washed with PBS containing 0.5% triton X-100. Finally, DNA staining was performed. Hoechst 33342 stain reagent was diluted with deionized water in a ratio of 100:1, and the cells were incubated with Hoechst 33342 stain (100 μL/well) for 30 min. The cells were imaged by 100× magnification. Images taken were processed and analyzed with software ImageJ.

### Reactive Oxygen Species–Level Detection

The cells in the six-well plate were subjected to different treatments as above. DCFH-DA (Beyotime, China) was diluted with DMEM in a ratio of 1:500. Forty-eight hours after the last treatment, 1 mL dilution solution was added to each well and incubated for 30 min. Then, the dilution solution was discarded, and the cells were collected for analysis with a FACS vantage SE flow cytometer (Becton Dickinson, San Jose, CA, United States) at an emission wavelength of 518 nm and an excitation wavelength of 494 nm.

### Detection of Apoptosis by Flow Cytometry

Human skin fibroblasts were uniformly scattered in six-well plates with the density of 2000/100 μL. Human skin fibroblasts were cultured for 48 h after different managements were done. Supernatant and HSFs detached from six-well plates were transferred to a new tube and centrifuged at 1000 *g* for 5 min. HSFs were then resuspended in the PBS twice to remove trypsin and covered with fluorescein isothiocyanate–labeled annexin V/PI (Vazyme, Nanjing, China) for 10 min. Then, the apoptosis rate was measured by flow cytometry.

### MiRNA Microarray

Total RNA was extracted using the mirVana RNA Isolation Kit (Applied Biosystems p/n AM1556) following the manufacturer’s recommendations. Cyanine-3 (Cy3) labeled RNA was prepared and purified from 100 ng of the diluted RNA using miRNA complete Labeling and Hyb Kit (Agilent) according to the manufacturer’s instructions. MiRNA complete Labeling and Hyb Kit and miRNA Spike-In Kit (Agilent) were used in process of hybridization following the manufacturer’s instructions. For microarray wash, Triton X-102 was added to Gene Expression wash buffers, and the Gene Expression wash buffer was prewarmed. Copious amounts of Milli-Q water were run through and emptied out the staining dish for at least five times, and then Milli-Q water was discarded for preparation of equipment. The microarray slides were washed with GE Wash Buffer. Slides were assembled into a slide holder, and the assembled slide holders were placed into scanner carousel. Slides were scanned on the Agilent Microarray Scanner (scan area 61 mm × 21.6 mm, scan resolution 5 μm, dye channel is set to green, and green PMT is set to 100%). Feature Extraction software (version 10.7.1.1, Agilent Technologies) was used to analyze array images to get raw data. Next, Genespring software (version 13.1, Agilent Technologies) was employed to finish the basic analysis with the raw data. The raw data were normalized with the quantile algorithm. The probes that at least 100% of samples in any one condition out of two conditions that have flags as “detected” were chosen for further data analysis. Differentially expressed miRNAs were then identified through fold change. Features flagged in Feature Extraction as feature non-uniform outliers were excluded. The data have been approved and deposited in the Gene Expression Omnibus (GEO accession no. GSE111444).

### qRT-PCR Analysis of miRNA and mRNA Expression Levels

MiRNA/mRNA was extracted with TRIzol reagent (Invitrogen, United States) following the manufacturer’s instructions. The qRT-PCR assay was conducted using a SYBR Green miRNA qRT-PCR quantitation kit protocol (Shanghai Integrated Biotech Solutions, Shanghai) according to the manufacturer’s instructions. MiRNA expression levels were tested by IQ5 Real-time PCR Instrument (Bio-Rad, United States). All RT-PCRs were performed in triplicate. In our research, the value of | log2(control/S-UV)| was considered as a significant change when it was ≥5. The relative expression of miRNAs was analyzed with 2^–ΔΔCt^ method using U6 for normalization (hsa-miR-8071, hsa-miR-769-5p, hsa-miR-758-3p, hsa-miR-7515, hsa-miR-6856-5p, hsa-miR-6837-5p, hsa-miR-6769a-5p, hsa-miR-6743-5p, hsa-miR-4694-3p, hsa-miR-4655-3p, hsa-miR-4514, hsa-miR-432-5p, hsa-miR-3163 and hsa-miR-3659, hsa-miR-4513, hsa-miR-4422, hsa-miR-1299, hsa-miR-564, and hsa-miR-22-5p) (Table 1).

**TABLE 1:** Real-time quantitative PCR primer sequence used in this study.

**miRNA**	**F primer**	**R primer**
hsa-miR-8071	GTCTGTTCGGTGGACTGGAGT	TATGCTTGTTCTCGTCTCTGTGTC
hsa-miR-769-5p	CTCTCTTGAGACCTCTGGGTTC	TATGCTTGTTCTCGTCTCTGTGTC
hsa-miR-758-3p	CTCTCTTTTGTGACCTGGTCCA	TATGCTTGTTCTCGTCTCTGTGTC
hsa-miR-7515	TGCTCTGTCAGAAGGGAAGAT	TATGCTTGTTCTCGTCTCTGTGTC
hsa-miR-6856-5p	TTGTATAAGAGAGGAGCAGTGGTG	TATGCTTGTTCTCGTCTCTGTGTC
hsa-miR-6837-5p	TTATTACCAGGGCCAGCAGG	TATGCTTGTTCTCGTCTCTGTGTC
hsa-miR-6769a-5p	TCGTCTATAGGTGGGTATGGAGG	TATGCTTGTTCTCGTCTCTGTGTC
hsa-miR-6743-5p	AAGGGGCAGGGACGGG	TATGCTTGTTCTCGTCTCTGTGTC
hsa-miR-4694-3p	ACCTCTAAGCAAATGGACAGGATA	TATGCTTGTTCTCGTCTCTGTGTC
hsa-miR-4655-3p	CTCTCTACCCTCGTCAGGTCC	TATGCTTGTTCTCGTCTCTGTGTC
hsa-miR-4514	CTCGTCTAACACAGGCAGGATT	TATGCTTGTTCTCGTCTCTGTGTC
hsa-miR-432-5p	TGCTCTAAGTCTTGGAGTAGGTCATT	TATGCTTGTTCTCGTCTCTGTGTC
hsa-miR-3163	CTTGCGAAACTGTATAAAATGAGG	TATGCTTGTTCTCGTCTCAGTGTC
hsa-miR-3659	CTGCTCTAACTGAGTGTTGTCTACG	TATGCTTGTTCTCGTCTCTGTGTC
hsa-miR-4513	CTCTAACAGACTGACGGCTGGAG	TATGCTTGTTCTCGTCTCTGTGTC
hsa-miR-4422	ACGTCTAAGAAAAGCATCAGGAAG	TATGCTTGTTCTCGTCTCTGTGTC
hsa-miR-1299	TCGTGTATTTCTGGAATTCTGTGT	TATGCTTGTTCTCGTCTCTGTGTC
hsa-miR-564	AACTTAAAAGGCACGGTGTCAG	TATGCTTGTTCTCGTCTCTGTGTC
hsa-miR-22-5p	TATAGTAGAAAGCTGCCAGTTGAAG	TATGGTTGTTCTGCTCTCTGTGTC

### Bioinformatics Analysis of Target Genes

Target genes of screened miRNA were predicted by data from TargetScan, microRNAorg, and PITA database with GeneSpring12.5 software.^1^ Results of the data were integrated for the correction. Gene Ontology (GO) analyses were used for functional classification of the targeted genes, which can be classified into genes biological process, molecular function, and cellular component. Pathways were analyzed by Kyoto Encyclopedia of Genes and Genomes (KEGG) pathways research.

### Transfection With the Synthetic miRNA Mimics, Inhibitor, and Negative Control

Mimic, inhibitor, and negative control (NC) of miR-769-5p were synthesized by Ribobio (Guangzhou, China). Quantitative reverse transcriptase–polymerase chain reaction method was used to measure the efficiency of transfection of HSF. Mimics and NC were diluted to a concentration of 50 and 100 nm, respectively, with buffer. Then, a certain dose of CP reagent and medium was added and incubated for 10 min on the ice. Previous culture medium was removed and replaced by the mixture. The HSF was incubated with the mixture for 48 h before the detection.

### Western Blotting

The HSFs cultured with exosomes-free medium for 48 h after transfection were lysed in RIPA buffer with added 1% phenylmethylsulfonyl fluoride (Beyotime, China). Protein supernatant was obtained by centrifugation after ultrasonic treatment. The protein concentration was determined using a Pierce BCA Protein Assay Kit (Beyotime, China). Proteins were separated by 10% SDS–polyacrylamide gel electrophoresis (Beyotime, China) and transferred to a methanol-activated polyvinylidene fluoride membrane (GE Healthcare, United States). The membrane was blocked for 1 h and half in TBST containing 5% milk and cleaned 5 min for three times in TBST. Subsequently, the membrane was probed with anti-B-actin (1:1000 dilution, Boorson, China)/anti-*TGFBR1* (1:100 dilution, Abcam, United Kingdom) antibody overnight at 4°C separately. Goat anti-rabbit immunoglobulin G (H + L) horseradish peroxidase (1:10,000 dilution) was used as secondary antibodies (Beyotime, China) for 1-h incubation. Finally, the protein bands were detected with Band-Scan software (PROZYME, San Leandro, CA, United States) after treatment with ECL reagents (Thermo Fisher Scientific).

### Dual-Luciferase Reporter Assay

Wild-type 3′-untranslated region (3′-UTR) and mutant sequences of *TGFBR1* at the predicted target region for miR-769-5p (5′ACTGAGGTTAGAGCTAGTGTGGTTTTGAGGTCTCA CTACACTT-TGAGGAAGGCAGCTTTT3′ and 5′ACTGAGG TTAGAGCTAGTGTGGTTTTGTCCAGAGAC-TACACTTTGA GGAAGGCAGCTTTT3′) were, respectively, cloned into the pmirGLO vector to obtain *TGFBR1* wild-type (WT) and *TGFBR1* mutant-type (MUT) constructs. The HSFs were seeded into 24-well plates. Approximately 24 h later, the cells were transfected with 800 ng experimental plasmid including *TGFBR1* WT and *TGFBR1* MUT and control plasmid. Five hours later, the medium was replaced, and the HSFs were transfected with 50 nm miR-769-5p mimic or mimic NC after 3-h incubation. After transfection for 48 h, 100 μL passive lysis buffer (Promega, United States) was used for preparation of cell lysates. The assays for firefly luciferase activity and Renilla luciferase activity were performed sequentially using one reaction tube. Finally, the firefly luciferase activity was normalized with Renilla luciferase.

### Statistical Analysis

Samples were determined in triplicate, and all experiments were repeated at least three times independently, whereas data are expressed as the mean ± SD for each group. Statistical analysis was performed using SPSS software (Windows version 16.0). Student *t* tests and χ^2^ test were used for statistical analysis. *P* < 0.05 was considered to be significant.

## Results

### MiRNA Expression Levels of Exosomes in Culture Medium Were Changed After UV Irradiation

**FIGURE 1:**
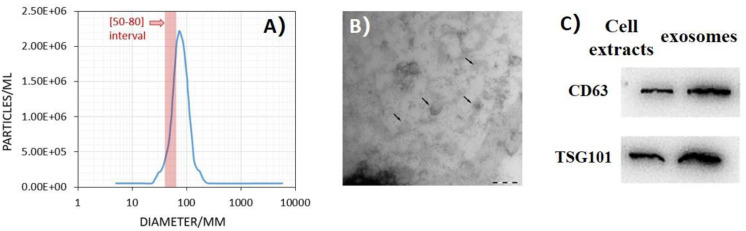
Ultraviolet radiation–induced bystander effects (UV-RIBEs) can be induced by exosome transfer. **(A)** The component showed a diameter from 50 to 80 nm with nanoparticle tracking analysis (NTA) method. **(B)** The exosomes were visualized as a typical tea tray-like structure with transmission electron microscopy. **(C)** Expression of typical proteins marker of exosome was higher than that in the cell protein group (*P* < 0.05).

The exosomes extracted from the conditioned medium were identified by NTA, TEM, and WB methods. To assess the size distribution of extracted components, we used NTA and found that the components had a diameter from 50 to 80 nm, in accordance with the characteristic size of exosomes (Figure 1A). As shown in Figure 1B, the exosomes were visualized as a typical tea tray-like structure with TEM. In addition, WB method was performed to confirm the expression of typical protein marker of exosomes. The results showed the expression of CD63 and TSG101 in the exosomes derived from conditioned medium (Figure 1C).

To screen differential expressed miRNAs in exosomes after UV irradiation, microarray method was used to detect the expression levels of miRNAs in exosomes. Differentially expressed miRNAs were identified with microarray and screened according to fold changes (more than five times). When comparing with exosomes in the culture medium of the control group, 54 miRNAs were upregulated, and 11 miRNAs were downregulated in that of the UVA group, whereas 50 miRNAs were upregulated, and four miRNAs were downregulated in that of the UVB group. Among all the differentially expressed miRNAs, 19 miRNAs were upregulated in exosomes in culture medium of both UVA and UVB groups (Table 2).

**TABLE 2:** MiRNA microarray results of upregulated miRNAs in exosomes ($\bar{X}\pm s$, *n* = 3).

**miRNA**	**Log FC ([A] vs. [O])**	**Log FC ([B] vs. [O])**
hsa-miR-8071	6.445 ± 1.081	6.110 ± 1.021
hsa-miR-769-5p	6.575 ± 1.134	6.427 ± 1.058
hsa-miR-758-3p	6.062 ± 0.998	6.552 ± 1.215
hsa-miR-7515	7.308 ± 1.268	6.609 ± 1.089
hsa-miR-6856-5p	6.144 ± 1.075	6.249 ± 1.019
hsa-miR-6837-5p	6.232 ± 1.109	6.942 ± 1.432
hsa-miR-6769a-5p	6.206 ± 1.091	6.277 ± 1.126
hsa-miR-6743-5p	6.345 ± 1.123	6.175 ± 1.110
hsa-miR-4694-3p	7.945 ± 1.978	6.027 ± 1.576
hsa-miR-4655-3p	6.753 ± 1.287	6.021 ± 1.012
hsa-miR-4514	6.136 ± 0.932	6.908 ± 1.210
hsa-miR-432-5p	7.320 ± 1.512	6.279 ± 1.313
hsa-miR-3163	6.424 ± 0.543	6.205 ± ± 0.495
hsa-miR-3659	6.070 ± 0.701	6.708 ± 0.712
hsa-miR-4513	6.794 ± 1.345	6.354 ± 1.011
hsa-miR-4422	7.774 ± 2.013	5.879 ± 0.835
hsa-miR-1299	6.205 ± 0.5341	6.330 ± 0.567
hsa-miR-564	6.674 ± 1.567	7.795 ± 2.013
hsa-miR-22-5p	6.668 ± 0.52	6.232 ± 0.432

Based on the results, qRT-PCR method was used further to detect expression level of these 19 miRNAs for data reliability. According to the result of qRT-PCR method, there were 13 miRNAs, which were upregulated in exosomes in culture medium of both UVA and UVB groups (hsa-miR-8071, hsa-miR-769-5p, hsa-miR-758-3p, hsa-miR-7515, hsa-miR-6856-5p, hsa-miR-6837-5p, hsa-miR-6769a-5p, hsa-miR-6743-5p, hsa-miR-4694-3p, hsa-miR-4655-3p, hsa-miR-4514, hsa-miR-432-5p, and hsa-miR-3163). Five miRNAs showed upregulation only in exosomes in culture medium of UVA group (hsa-miR-4513, hsa-miR-4422, hsa-miR-1299, hsa-miR-3659, and hsa-miR-564), and hsa-miR-22-5p was downregulated in that of the UVA and UVB groups. Results of qRT-PCR method were consistent with microarray results basically (Table 3).

**TABLE 3:** qRT-PCR results of upregulated miRNA in exosomes ($\bar{X}\pm s$, *n* = 3).

**miRNA**	**Control group**	**UVA group**	**UVB group**
hsa-miR-8071	1 ± 0.075	3.676 ± 0.542	3.200 ± 0.674
hsa-miR-769-5p	1 ± 0.234	2.971 ± 0.352	2.686 ± 0.501
hsa-miR-758-3p	1 ± 0.113	4.622 ± 0.596	7.027 ± 0.467
hsa-miR-7515	1 ± 0.065	3.884 ± 0.310	5.345 ± 0.793
hsa-miR-6856-5p	1 ± 0.056	6.5 ± 1.174	2.677 ± 0.592
hsa-miR-6837-5p	1 ± 0.056	2.972 ± 0.562	3.401 ± 0.126
hsa-miR-6769a-5p	1 ± 0.081	2.669 ± 0.296	1.623 ± 0.255
hsa-miR-6743-5p	1 ± 0.108	3.909 ± 0.437	3.568 ± 0.530
hsa-miR-4694-3p	1 ± 0.065	3.242 ± 0.224	3.519 ± 0.328
hsa-miR-4655-3p	1 ± 0.038	3.191 ± 0.311	5.517 ± 0.273
hsa-miR-4514	1 ± 0.037	3.436 ± 0.193	1.948 ± 0.269
hsa-miR-432-5p	1 ± 0.047	4.053 ± 0.674	2.766 ± 0.593
hsa-miR-3163	1 ± 0.228	4.313 ± 1.066	1.877 ± 0.425
hsa-miR-3659	1 ± 0.125	1.538 ± 0.246	1.115 ± 0.352
hsa-miR-4513	1 ± 0.071	1.448 ± 0.245	0.662 ± 0.113
hsa-miR-4422	1 ± 0.141	3.097 ± 0.259	0.962 ± 0.110
hsa-miR-1299	1 ± 0.301	2. ± 0.789	0.493 ± 0.242
hsa-miR-564	1 ± 0.198	2.439 ± 0.299	0.843 ± 0.176
hsa-miR-22-5p	1 ± 0.023	0.384 ± 0.015	0.107 ± 0.028

**FIGURE 2:**
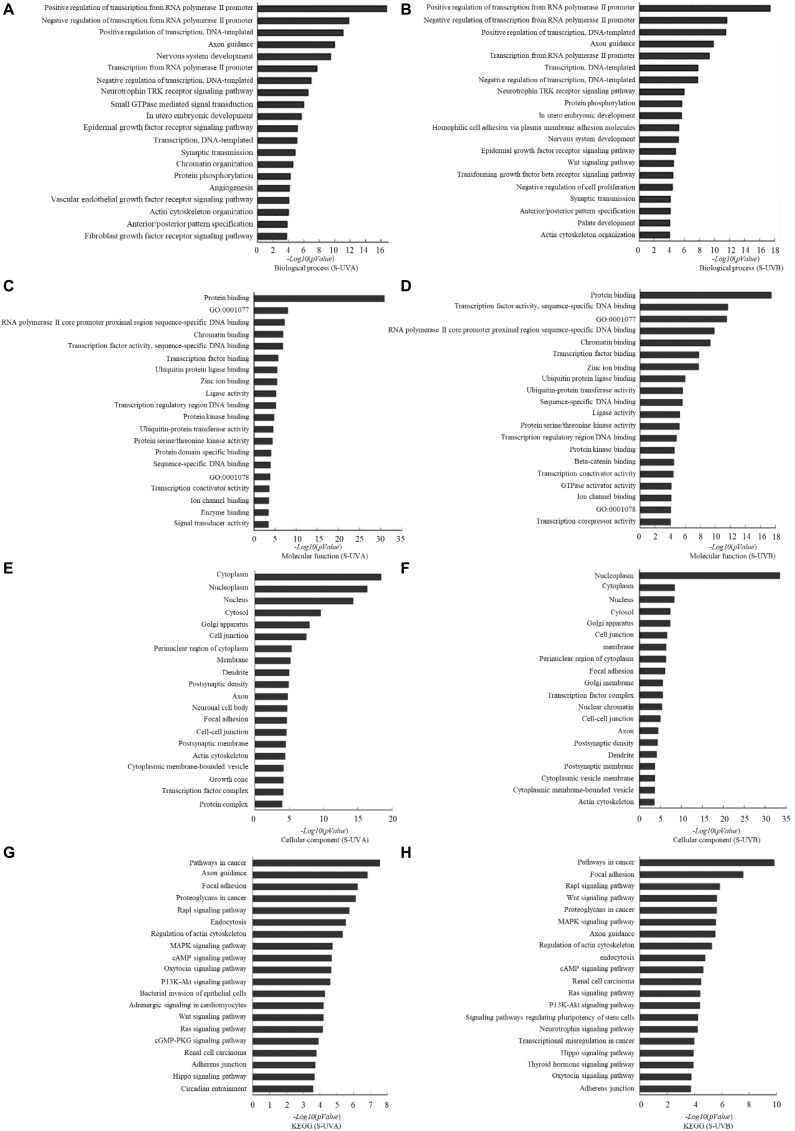
Target gene prediction and bioinformatics analysis based on TargetScan, microRNAorg, PITA databases: differentially expressed exosomal miRNAs–involved biological process indicated by GO analysis in UVA group **(A)** and UVB **(B)** group. Differentially expressed exosomal miRNAs–involved molecular function indicated by GO analysis in UVA group **(C)** and UVB group **(D)**. Differentially expressed exosomal miRNAs–involved cellular component indicated by GO analysis in UVA group **(E)** and UVB group **(F)**. Differentially expressed exosomal miRNAs–involved KEGG pathway in UVA group **(G)** and UVB group **(H)**.

Based on the result of miRNA screening, relative target genes were predicted according to TargetScan, PITA, and microRNAorg databases. According to the intersection of the three databases, the common target genes were analyzed for the biological processes, molecular functions, and cellular components with GO analysis and KEGG analysis. Biological processes that potentially participated by target genes included regulation of transcription, axon guidance, nervous system development, neurotrophin TRK receptor signaling pathway, and epidermal growth factor receptor signaling pathway. In molecular function, it showed that those target genes were involved in binding of protein, RNA polymerase II core promoter proximal region sequence-specific DNA, and chromatin and transcription factor. Cellular components included cytoplasm, nucleoplasm, nucleus, cytosol, Golgi apparatus, and cell junction. Kyoto Encyclopedia of Genes and Genomes analysis showed that those target genes played roles in pathway of cancer, focal adhesion, Rap1 signaling pathway, proteoglycans in cancer, MAPK signaling pathway, etc. (Figure 2).

### Exosome Transfer Can Induce UV-RIBEs and Change the miRNA Expression Levels in Bystander Cells

**FIGURE 3:**
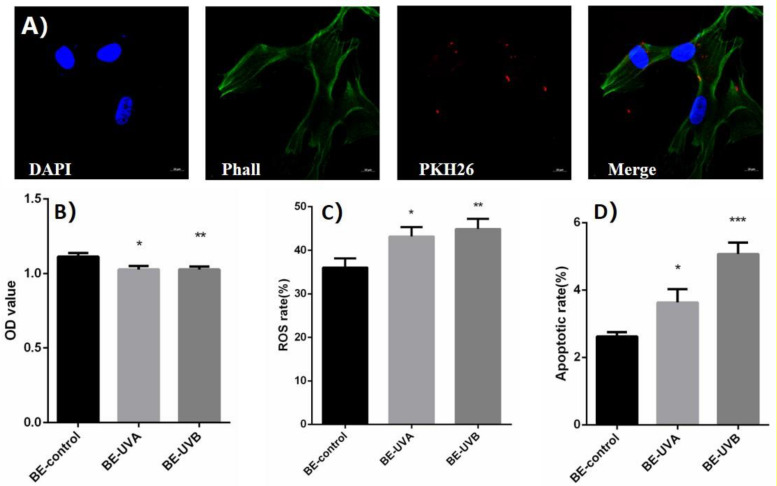
**(A)** The spotted red fluorescence in the cytoplasm of human skin fibroblasts (HSFs) was observed under laser light focusing microscope. **(B)** Optical density (OD) value of exosome transferred bystander cells in UVA/B-irradiated group. **(C)** Reactive oxygen species (ROS) level of exosome transferred bystander cells in UVA/B-irradiated group (DCFH-DA single-staining method). **(D)** Apoptosis rate of exosome transferred bystander cells in UVA/B-irradiated group. *P < 0.05 comparing with BE (bystander effect)–control group. **P < 0.01 comparing with BE-control group. ***P < 0.001 comparing with BE-control group.

The exosomes extracted from conditioned medium were labeled with PKH26 and coincubated with normal HSFs. Spotted red fluorescence in the cytoplasm of HSFs was observed under laser light focusing microscope, indicating that the exosomes added to the culture medium can be ingested by normal HSFs, shown in Figure 3A.

After being cocultured with extracted exosomes for 48 h, biological indicators of bystander HSFs were detected. CCK-8 was performed and showed that the cell proliferation activity of bystander HSFs treated with the exosomes from irradiated group decreased significantly (Figure 3B). Figure 3C showed that compared to BE (bystander effect)–control group, the fluorescence intensity of DCF in the bystander group was significantly increased, indicating an increased reactive oxygen species (ROS) level, whereas the apoptotic rates of bystander HSFs in the BE-UVA group and the BE-UVB group were significantly higher than that in the BE-control group as shown in Figure 3D.

**FIGURE 4:**
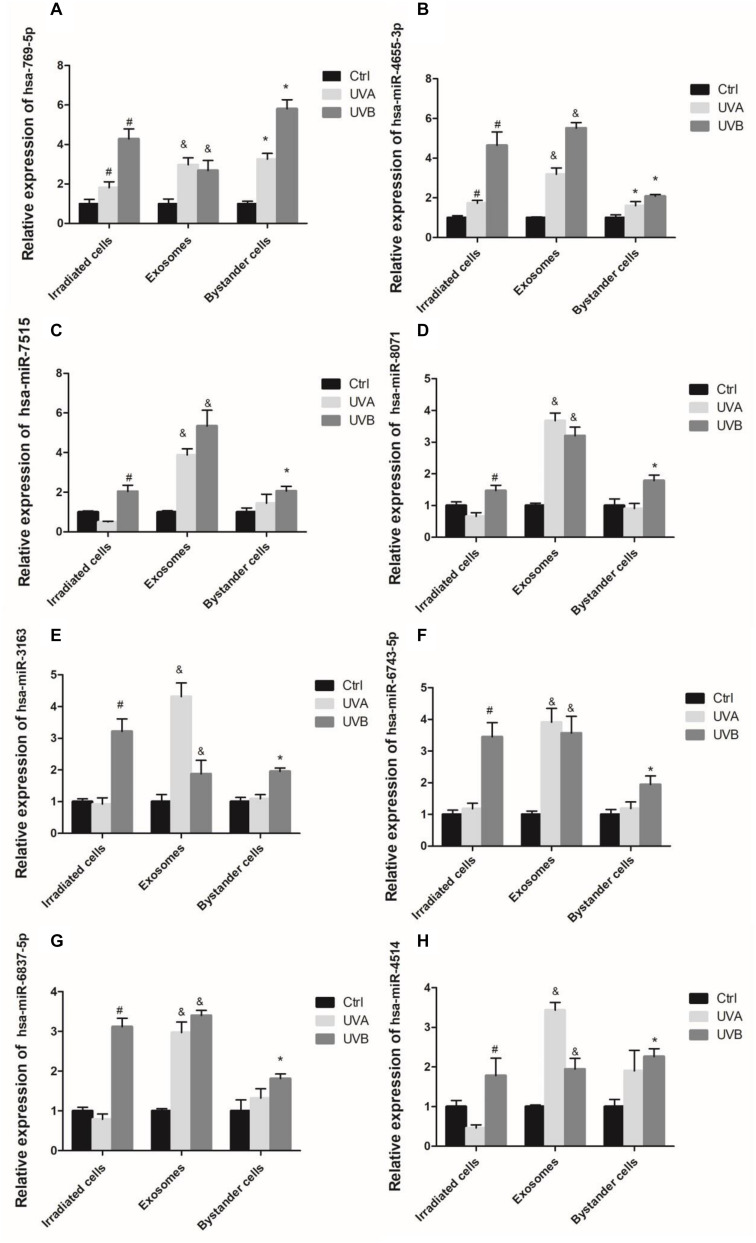
Expression levels of exosomal miRNAs measured by qRT-PCR in UV-RIBEs, including miR-769-5p **(A)**, miR4655-3p **(B)**, miR-7515 **(C)**, miR-8071 **(D)**, miR-3163 **(E)**, miR-6743-5p **(F)**, miR-6837-5p **(G)**, and miR-4514 **(H)**. ^#^*P* < 0.05 as compared with control group. ^&^*P* < 0.05 comparing with exosome of control group. *P < 0.05 as compared with bystander control group.

Expression levels of miRNA in irradiated cells and bystander cells were tested by the qRT-PCR method subsequently. Among the 13 miRNAs mentioned above, 8 miRNAs showed an increasing level of expression in both irradiated HSFs and bystander HSFs. In all the eight miRNAs, miR-4655-3p and miR-769-5p had a significant overexpression in both UVA-irradiated and BE-UVA HSFs, as well as in UVB-irradiated HSFs and BE-UVB HSFs. The rest showed an upregulation in UVB-irradiated and BE-UVB HSFs, but there was no significant difference between UVA-irradiated HSFs and BE-UVA HSFs (including miR-8071, miR-7515, miR-6837-5p, miR-6743-5p, miR-4514, and miR-3163) as shown in Figure 4.

### MiR-769-5p Was Transferred Between Irradiated HSFs and Bystander HSFs in UV-RIBEs

**FIGURE 5:**
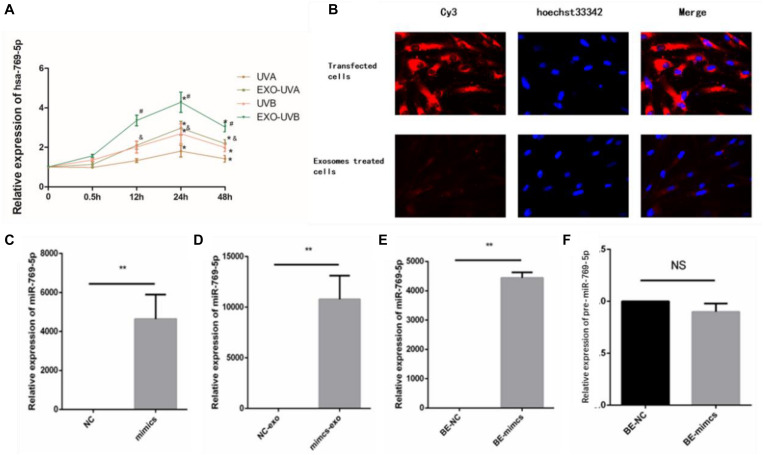
MiR-769-5p was transferred between irradiated HSFs and bystander HSFs in UV-RIBE. **(A)** Time-dependent expression tendency of miR-769-5p in irradiated HSFs (UVA/UVB group) and exosomes (EXO-UVA/UVB group) after irradiation. *P < 0.05 comparing with BE-control group. **(B)** The miR-769-5p–labeled red fluorescence of Cy3 was observed both in transfected HSFs and bystander cells, indicating miRNA shuttling through exosomes. **(C)** The expression of miR-769-5p in mimics group after transfection. **P < 0.01 comparing with negative control (NC) group. **(D)** The expression of miR-769-5p in exosomes after transfection. **P < 0.01 comparing with NC group. **(E)** The expression of miR-769-5p in bystander cells after transfection. **P < 0.01 comparing with NC group. **(F)** Expression level of miR-769-5p precursor (pre-miR-769-5p) in bystander cells. NS, no statistical difference.

We analyzed time-dependent expression of miR-769-5p in irradiated HSFs and secreted exosomes in culture medium after UVA and UVB irradiation with qRT-PCR method. The expression of miR-769-5p in exosomes of UVA- and UVB-irradiated HSFs started to increase significantly at 12 h after irradiation. Expression of miR-769-5p in UVA-irradiated HSFs and UVB-irradiated HSFs showed a significant increase at 24 h after irradiation. It took 24 h to reach the maximum expression in both of irradiated HSFs and secreted exosomes. An apparent higher expression level of miR-769-5p in exosomes than irradiated HSFs was observed at 12 and 24 h after irradiation (Figure 5A). In order to verify that miR-769-5p can shuttle between cells by exosomes, we transfected normal HSFs with miR-769-5p with Cy3 fluorescence and extracted exosomes from the culture medium 24 h after transfection. The exosomes were added to normal HSFs for coincubation. During this process, direct transfected HSFs and the HSFs treated with extracted exosomes were observed by fluorescence microscopy, and red fluorescence was observed both in transfected HSFs and the HSFs treated with exosomes extracted, labeled by Cy3. The labeled miR-769-5p was successfully transfected into HSFs and then encapsulated into exosomes and shuttle into bystander HSFs through exosomes, shown in Figure 5B.

In order to identify the transfer of miR-769-5p, qRT-PCR was performed to detect miR-769-5p mimic transfected cells (mimics group), secreted exosomes (mimics-exo group), and exosome-incubated bystander cells (BE-mimics group). There was a significant increase in the expression of miR-769-5p in mimics group when being compared with NC group, shown in Figure 5C. The same tendency could also be found in mimics-exo group and BE-mimics group (Figures 5D,E). In addition, no statistical difference of expression level of miR-769-5p precursor (pre-miR-769-5p) was observed between BE-mimics group and BE-NC group (Figure 5F).

### MiR-769-5p Can Induce Bystander-Like Effects in HSFs

**FIGURE 6:**
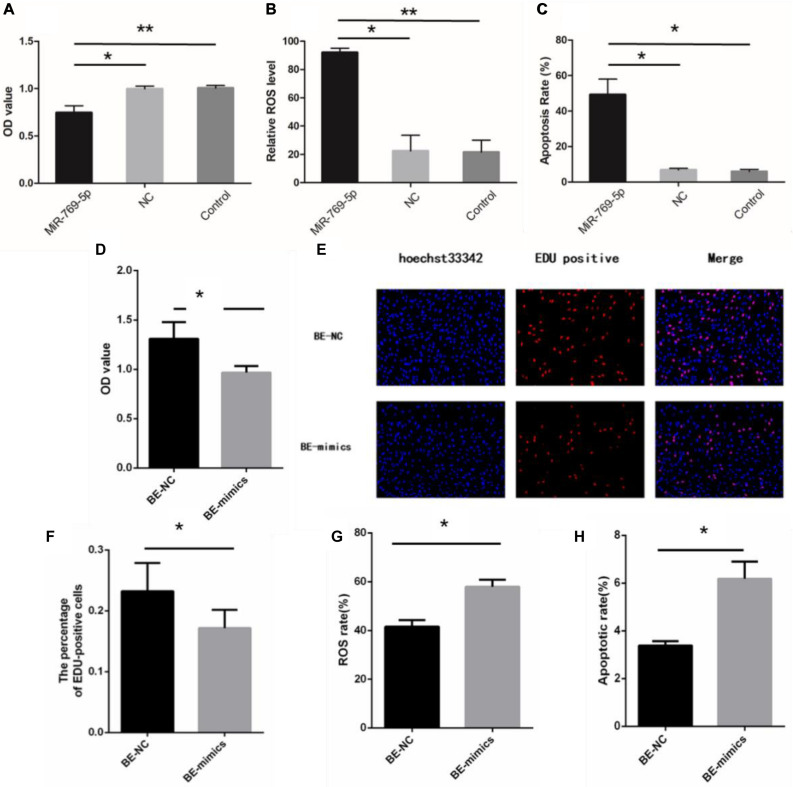
Upregulation of miR-769-5p can induce UV-RIBEs. **(A)** Cell proliferation rate of HSFs after treatment with miR-769-5p mimic and negative control (NC). **(B)** Oxidative damage level of HSFs after treatment with miR-769-5p mimic and NC. **(C)** Apoptosis rate of HSFs after treatment with miR-769-5p mimic and NC. **(D)** Cell proliferation rate of bystander HSFs with or without miR-769-5p mimic treatment. **(E)** EdU staining of bystander HSFs with or without miR-769-5p mimic treatment. **(F)** Percentage of EdU-positive cells in bystander HSFs with or without miR-769-5p mimic treatment. **(G)** Oxidative damage level of bystander HSFs with or without miR-769-5p mimic treatment. **(H)** Apoptosis rate of bystander HSFs with or without miR-769-5p mimic treatment. *P < 0.05 comparing with control group, **P < 0.01 comparing with control group.

After being transfected with miR-769-5p mimics, HSFs displayed a decrease of the OD value compared with the control group and NC group (Figure 6A). Besides, HSFs transfected with miR-769-5p mimics had a remarkable increase of ROS level and higher level of cell apoptosis (Figures 6B,C). Subsequently, we examined the biological indicators of HSFs incubated with exosomes from high expression of miR-769-5p for 48 h. Figure 6D showed that the cell proliferation activity in the BE-NC group was higher than that in the BE-mimics group, with statistical significance (*P* < 0.05). The experiment of EdU staining also supported this trend (Figures 6E,F). Comparing to BE-NC group, the oxidative damage level and apoptotic rate in the BE-mimics group were significantly increased (Figures 6G,H).

**FIGURE 7:**
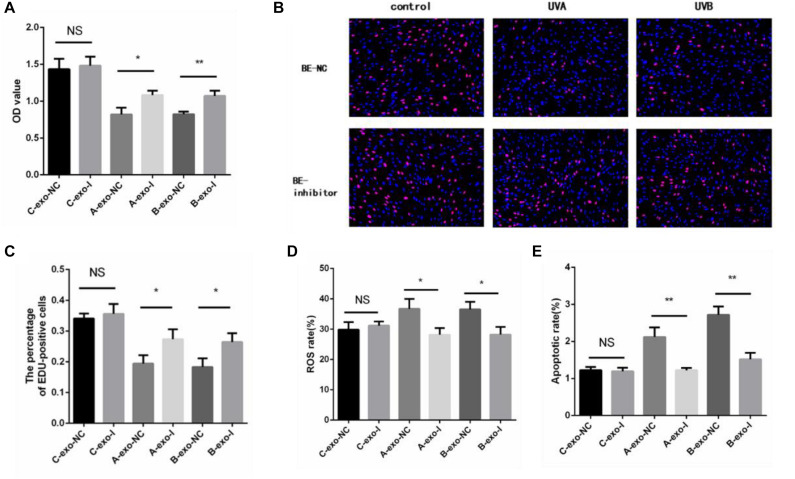
Downregulation of miR-769-5p can suppress UV-RIBEs. **(A)** Cell proliferation level of NC-/inhibitor-transfected bystander HSFs after being treated with exosomes from UVA/B-irradiated HSFs and non-irradiated HSFs. **(B)** EdU staining of NC-/inhibitor-transfected bystander HSFs after being treated with exosomes from UVA/B-irradiated HSFs and non-irradiated HSFs. **(C)** Percentage of EdU-positive cells in negative control (NC)-/inhibitor (I)–transfected bystander HSFs after being treated with exosomes (exo) from UVA/B-irradiated HSFs and non-irradiated HSFs. (A: UVA irradiated group, B: UVB irradiated group, C: control group) **(D)** ROS level of NC-/inhibitor-transfected bystander HSFs after being treated with exosomes from UVA/B-irradiated HSFs and non-irradiated HSFs. **(E)** Apoptosis rate of NC-/inhibitor-transfected bystander HSFs after being treated with exosomes from UVA/B-irradiated HSFs and non-irradiated HSFs. *P < 0.05 comparing with control group, **P < 0.01 comparing with control group. NS, no statistical difference.

In addition, we detected the proliferation rate, oxidative damage, and apoptosis rate in non-irradiated HSFs by exosomes with or without miR-769-5p inhibitor. Figure 7A showed that the cell proliferation level of inhibitor-transfected bystander HSFs was higher than NC-transfected bystander HSFs after being coincubated with exosomes from UVA/UVB-irradiated HSFs. EdU staining showed a stronger fluorescent intensity in inhibitor-transfected bystander HSFs than in NC-transfected bystander HSFs (Figures 7B,C). After being treated with exosomes from UVA/UVB-irradiated HSFs, a higher level of ROS was observed in NC-transfected bystander HSFs than inhibitor-transfected bystander HSFs (Figure 7D), whereas the apoptosis rate of NC-transfected bystander HSFs also increased remarkably when compared with inhibitor-transfected bystander HSFs (Figure 7E).

### MiR-769-5p Downregulated TGFBR1 Expression by Targeting the TGFBR1 3′-UTR

**FIGURE 8:**
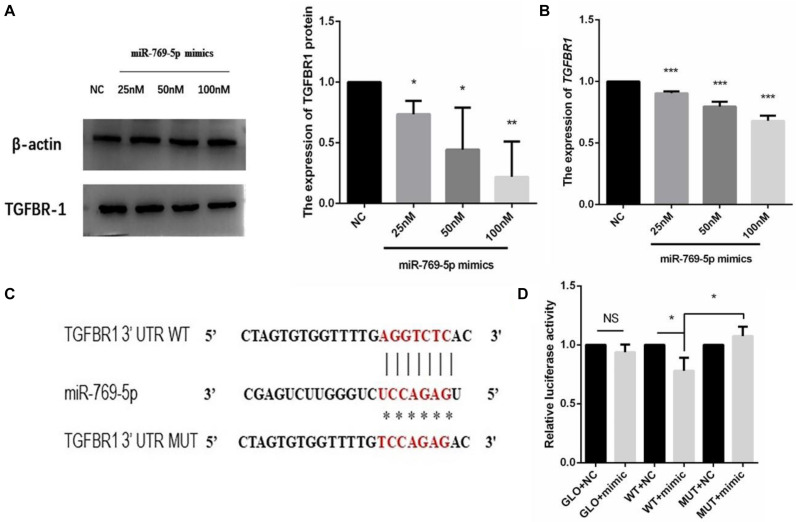
Search and identification of the downstream target genes of miR-769-5p. **(A)** Protein expression of *TGFBR1* in HSFs after transfection of miR-769-5p mimic from 25 to 100 nM. **(B)** MRNA expression of *TGFBR1* in HSFs after transfection of miR-769-5p mimic from 25 to 100 nM. *P < 0.05 comparing with NC group. **P < 0.01 comparing with NC group. ***P < 0.001 comparing with NC group. **(C)** Target site of miR-769-5p in *TGFBR1* mRNA 3′-UTR was predicted and luciferase report vector of relative wild and mutant type were built. **(D)** Fluorescence activity of wild type and mutant type after transfection of miR-769-5p mimic. GLO, pmirGLO vector; WT, wild type; MUT, mutant type. *P < 0.05. NS, no statistical difference.

The target gene of miR-769-5p, *TGFBR1*, was predicted with software such as TargetScan and DIANA Tools, combined with previous researches. The qRT-PCR and WB results showed that the transfection of miR-769-5p mimic suppressed the mRNA and protein expression of *TGFBR1* in HSFs, and the expression level of *TGFBR1* decreased in accordance with the increasing concentration of miR-769-5p mimic (Figures 8A,B). In order to verify the effect of miR-769-5p on *TGFBR1* mRNA 3′-UTR, target site of miR-769-5p in *TGFBR1* mRNA 3′-UTR was predicted through TargetScan (Figure 8C). Luciferase reporter vector containing the sequences of the *TGFBR1* mRNA 3′-UTR and its mutant were constructed separately. Transfection of miR-769-5p mimic decreased the luciferase activity of *TGFBR1* 3′-UTR vector, whereas there was no influence on the *TGFBR1* 3′-UTR mutant (Figure 8D).

## Discussion

Earlier experiments elucidated that transportation of intercellular substance acts as an important mediator in bystander effects, including cytokines, ROS, and extracellular vesicles, inducing oxidative damage and apoptosis. In this study, exosomal miRNAs in culture medium were screened and identified with the technology of microarray and qRT-PCR after UV irradiation. The results indicated that a number of miRNAs were differentially expressed in UV-RIBEs, and miR-769-5p was found upregulated in culture medium and irradiated and bystander HSFs. Shuttle of miR-769-5p between transfected HSFs and bystander HSFs was observed. Moreover, HSFs transfected with miR-769-5p mimics showed a decrease in proliferation, along with increases in ROS level and apoptosis, and target gene *TGFBR1* was verified subsequently. Our findings demonstrated that exosomal miR-769-5p may play a critical role in UV-RIBEs by targeting *TGFBR1*. This research has investigated the potential molecular mechanisms of UV-RIBEs and may serve for further defining theory basis of bystander effect in UV irradiation.

Previous researches indicated an important role of exosome-mediated miRNAs in bystander effect, which are transported to and accepted by recipient cells, inducing signal communication through cell membrane, altering gene expression as cellular miRNAs (Kosaka et al., 2010; Xu et al., 2015). However, it has been shown that miRNAs are not randomly incorporated into exosomes. Mo et al. (2018) demonstrated that miR-1246 packaged in exosomes from γ-ray-irradiated BEP2D cells can cause DNA damage in bystander cells by targeting LIG4 gene. Exosomes-containing miR-7-5p was observed to induce bystander autophagy (Song et al., 2016). Thus, we assumed that specific miRNAs may also take part in the mechanism of UV-RIBEs. In this research, secretory miRNAs packaged in exosomes were investigated. Through the analysis of miRNAs in cultured medium by microarray, differentially expressed miRNAs were preliminary screened. To explore the potential biological processes and pathways, target genes of those differentially expressed miRNAs were predicted by integrated results from TargetScan, PITA, and microRNAorg databases. A set of biological processes and pathways was predicted according to GO analysis and KEGG pathway. Predicted biological processes were mostly enriched in energy production, transcription regulation, transmembrane signal, and cell proliferation. Metabolic pathways included focal adhesion kinase (FAK)-related signaling pathways and MAPK signaling pathway. According to previous study, FAK signaling pathways and MAPK signaling pathways take the roles in a variety of cellular processes such as proliferation, apoptosis, and oxidative damage (Wang et al., 2015; Yang et al., 2016).

Expression levels of miRNAs in irradiated cells, culture medium, and recipient bystander cells were detected by qRT-PCR method to demonstrate the intercellular transfer of exosomal miRNAs in UV-RIBEs. Upregulation of miR-769-5p in irradiated HSFs, cultured medium, and recipient bystander HSFs indicated a potential transfer of miRNA between irradiated cells and bystander cells, which is in line with previous studies. For further study, we labeled transfected miR-769-5p with Cy3 and tracked exosome-mediated miR-769-5p shuttle between transfected HSFs and bystander HSFs. Expression levels of miR-769-5p in transfected HSFs, exosomes, and bystander HSFs were detected after transfection and coincubation. Upregulation of miR-769-5p was found in both exosomes and bystander HSFs, demonstrating an exosome-mediated miRNA shuttle.

MiR-769-5p was found upregulated in hypoxia-induced human lung adenocarcinoma cells, affecting the cell cycle of A549 cells, while its predicted target genes, including ARID1A and SMAD2, were downregulated (Geng et al., 2016). SMAD2, participating in TGFβ1-Smad2 pathway, has been demonstrated to be involved in initiation of bystander effect in irradiated HaCaT cells (Yin et al., 2015). It was also found that miR-769-5p inhibited cell proliferation and migration in A549 and H157 cell lines by targeting *TGFBR1* (Yang et al., 2017). Those researches indicated that miR-769-5p has some effects on cell proliferation and oxidative damage through activating signaling pathway. We speculate that secretory miRNAs taken by recipient bystander HSFs may affect biological processes in the HSFs via targeting related genes in UV-RIBEs. As expected, upregulation of miR-769-5p in HSFs caused photodamage-like phenomenon after transfection of miR-769-5p. Similar phenomenon was also observed in bystander HSFs after coincubation with exosomes from transfected cells. By contrast, inhibiting the expression of miR-769-5p could reduce photodamage in bystander HSFs after coincubation with exosomes from UVA/UVB-irradiated cells, demonstrating the role of miR-769-5p in photodamage and UV-RIBEs. To explore the specific mechanism of miR-769-5p, considering the research results and our previous studies, we performed the study on the relationship between exosomes-mediated miR-769-5p and its target genes. It was detected that there was a lower expression of *TGFBR1* after upregulating the expression of miR-769-5p in HSFs. Combined with the fluorescence activity results of WT and mutant type, we speculated that exosome-mediated miR-769-5p could inhibit the expression of *TGFBR1* by effecting *TGFBR1* mRNA 3′-UTR directly and influence TGF-β/Smad pathway.

Taken together, our experiment demonstrated that miR-769-5p could shuttle from irradiated HSFs to bystander HSFs, as an important molecular mechanism of UV-RIBEs and may be associated with inhibiting the expression of the target gene, *TGFBR1*. However, the mechanism of exosome-mediated transfer and miRNAs functions in RIBEs is complicated; more researches are still needed to be done. First, although a research on exosomal miRNAs *in vitro* was carried out in this study, research *in vivo* is still needed. Second, the way exosomal miRNAs regulate bystander effects through targeting downstream gene requires further study. Our research may provide new insight into the development of UV protection and treatment of UV-related diseases.

## Conclusion

UVA and UVB irradiation-induced bystander effect was observed by the means of exosomes transfer. MiRNAs in exosomes participating in this biological process were screened, and miR-769-5p was proved to play a role in the process of UV-RIBEs. Downstream gene *TGFBR1* was suppressed by miR-769-5p through targeting *TGFBR1* 3′-UTR. Our findings may contribute to further research on bystander effect in UVA and UVB irradiation and provide ideas for clinical anti-photodamage treatments. miR-769-5p was found to downregulate expression by directly targeting its 3′-UTR.

## Data Availability Statement

The datasets presented in this study can be found in online repositories. The names of the repository/repositories and accession number(s) can be found below: https://www.ncbi.nlm.nih.gov/geo/, GSE111444.

## Author Contributions

DL and BZ: conceptualization and supervision. NN, WM, and YT: formal analysis, investigation, and writing – original draft. DL and BZ: funding acquisition and project administration. JL and BZ: methodology. JC: software. JW: validation. HH: visualization. NN: writing – review and editing. All authors contributed to the article and approved the submitted version.

## Conflict of Interest

The authors declare that the research was conducted in the absence of any commercial or financial relationships that could be construed as a potential conflict of interest.

## Footnotes

1 http://www.targetscan.org, http://www.microrna.org/, http://genie.weizmann.ac.il/pubs/mir07
